# Effects of Perceived Neighborhood Characteristics and Use of Community Facilities on Physical Activity of Adults With and Without Disabilities

**Published:** 2010-08-15

**Authors:** Keith Christensen, Judith M. Holt, Justin F. Wilson

**Affiliations:** Department of Landscape Architecture and Environmental Planning; Utah State University, Logan, Utah; Utah State University, Logan, Utah

## Abstract

**Introduction:**

Using data from the 2004 Texas Behavioral Risk Factor Surveillance System, we investigated whether the physical activity behaviors of people with disabilities are related to their perceptions of the characteristics of the built environment and whether this relationship differs from that of people without disabilities.

**Methods:**

The research questions were, "Are perceived neighborhood characteristics and reported use of community facilities associated with reported leisure-time physical activity for adults aged 18 to 64 years with disabilities?"; "Are perceived neighborhood characteristics and reported use of community facilities associated with reported moderate to vigorous physical activity for adults with disabilities?"; and "To what extent do perceived neighborhood characteristics, reported use of community facilities, reported leisure-time physical activity, and reported moderate to vigorous physical activity differ between adults with disabilities and without disabilities?" We used logistic regression to analyze the responses.

**Results:**

People with disabilities were less likely to engage in leisure-time physical activity and meet recommendations for physical activity than people without disabilities. Participation of people with disabilities in leisure-time physical activity had significant correlations with positive perceptions of neighbors, physical activity, trails, parks, playgrounds, or sports fields, and with their use of private or membership-only recreation facilities. The presence of sidewalks was significantly related to whether people with disabilities met recommended levels of physical activity.

**Conclusion:**

Although people with disabilities engaged in less leisure-time physical activity and physical activity than people without disabilities, perceptions of the built environment and use of community facilities similarly affected people with and without disabilities.

## Introduction

Although people with disabilities represent 15% of the total US population (1), research on the effect of the built environment on physical activity among people with disabilities is limited (2). However, evidence suggests that the aspects of the built environment that encourage physical activity among the general population may facilitate physical activity in disabled populations (3). Few studies have assessed the effect of the built environment on physical activity behaviors of people with disabilities. These studies have primarily analyzed accessibility to recreation programs and fitness facilities (2,4), environmental supports that affect physical activity (3,5), and community mobility as influenced by the built environment (6). The purposes of this study are to investigate whether the perceptions of people with disabilities about characteristics of the built environment are related to their physical activity behaviors and whether this relationship differs from that of people without disabilities. Our hypothesis is that accessibility barriers in the built environment give people with disabilities less positive perceptions of the built environment that cause them to engage in less physical activity than people without disabilities.

Extensive epidemiologic studies have demonstrated strong associations between physical activity and health. Physical activity reduces the risk of many of the major causes of illness or death in the United States, such as cardiovascular disease, high blood pressure, obesity, and depression (7). Many studies have concluded that well-designed built environments encourage physical activity. Heath et al (8) identified 13 cross-sectional studies published between 1993 and 2003 that support community-scale urban design and land-use policies and practices that increase physical activity, such as walking and biking infrastructure, proximity to recreation areas, and the aesthetic and safety aspects of the street-scale built environment. More recent research indicates that the following factors affect health-related physical activity: urban form (9-11), neighborhood design (12,13), neighborhood environmental quality (14), street type (15), vegetation (16), the proportion of green space, residential density, and the general impression of activity-friendliness of the neighborhood (17). Because physical activity is significantly affected by the built environment, "how we design the built environment may hold tremendous potential for addressing many of the nation's greatest current public health concerns" (18).

The design of the built environment disproportionately affects people with disabilities in comparison with their peers (19,20). Conditions in the built environment may create barriers to people with disabilities that reduce opportunities to engage in physical activity (2,5,21,22) and contribute to disparities that persist in nearly every aspect of health among people with disabilities (23). In addition to improperly implemented regulations of the Americans with Disabilities Act, features such as pathway texture, disconnected pedestrian ways, signage, and slope have more influence on the participation of people with disabilities in physical activity than on people without disabilities (5). *Healthy People 2010* recognizes this health disparity and reports that 56% of adults with disabilities do not engage in leisure-time physical activity (LTPA) compared with 36% of people without disabilities (24). The prevalence of sedentary behaviors among people with disabilities increases their susceptibility to chronic diseases and secondary health conditions (25). Thus, *Healthy People 2010* includes a developmental objective to increase LTPA participation among people with disabilities by reducing environmental barriers (24).

## Methods

### Theoretical framework

This study follows an ecological approach to public health research, which examines the contribution of structural and environmental factors to health disparities. A socioecologic framework describes the influence of the built environment on health behavior through macropolicy and environmental processes that lead to differential access to community resources. Disadvantaged community members often lack access to health-promoting environments and programs (26).

We investigated the relationships between perceived neighborhood characteristics, use of community facilities, LTPA, and moderate to vigorous physical activity behaviors of people with disabilities compared with people without disabilities. The study participants were adults aged 18 to 64 years who lived in Texas and participated in the 2004 Texas Behavioral Risk Factor Surveillance System (BRFSS) survey (27). The Utah State University institutional review board approved the study design.

The 3 research questions were the following:

1. Are perceived neighborhood characteristics and reported use of community facilities associated with reported LTPA for adults with disabilities?
2. Are perceived neighborhood characteristics and reported use of community facilities associated with reported moderate to vigorous physical activity for adults with disabilities?
3. To what extent do perceived neighborhood characteristics, reported use of community facilities, reported LTPA, and reported moderate to vigorous physical activity differ for adults with and without disabilities?

This study resembles a 2009 study conducted by Velasquez et al (28) that used 2004 Texas BRFSS data to investigate the relationship of perceived neighborhood characteristics, use of community facilities, LTPA, and physical activity as reported by adults. In that study, data were stratified by sex, not by disability status.

Administered annually, the BRFSS collects and tracks health trends and risks factors nationally through a telephone survey of adults aged 18 years or older. In addition to the BRFSS core questions administered nationwide, the 2004 BRFSS survey conducted in Texas included 2 state-added modules: *Neighborhood*, designed to gather respondents' perceptions of their neighborhood environment and their use of recreational community facilities, and *Physical Activity*, to collect data regarding moderate and vigorous physical activity. The total sample of Texas BRFSS 2004 respondents (N = 6,317) was reduced to exclude respondents 65 years or older and those who did not provide age-related information (n = 1,209). People whose disability status was unknown (n = 161) were also excluded from the sample, resulting in a sample size of 4,947 adult residents of Texas aged 18 to 64 years. The response rate for the 2004 Texas BRFSS was 43% (29).

### Measures

Disability status was determined as a yes response to either of 2 BRFSS core questions: "Are you limited in any activities because of physical, mental, or emotional problems?" and "Do you now have any health problem that requires you to use special equipment, such as a cane, a wheelchair, a special bed, or a special telephone?"

In addition to disability, included demographic variables were age, education (less than high school graduate, high school graduate or some college, and college graduate), annual income (<$25,000, $25,000 to <$75,000, and $75,000 or more as aggregated in the BRFSS data), sex, and race/ethnicity (white, black, Hispanic, and other).

Respondents were reported to participate in LTPA if they answered yes to the following BRFSS core question: "During the past month, other than your regular job, did you participate in any physical activities or exercises such as running, calisthenics, golf, gardening, or walking for exercise?" As part of the state-added module *Physical Activity*, respondents were reported to meet recommended levels of physical activity based on self-reported participation in vigorous or moderate activities, described as follows: "Vigorous activities cause large increases in breathing or heart rate while moderate activities cause small increases in breathing or heart rate" (27). Respondents then reported days per week and minutes per day spent performing vigorous activities and moderate activities. Respondents were reported to meet recommended weekly levels of physical activity if they indicated at least 3 days with 20 minutes of vigorous activity or 5 days with 30 minutes of moderate activity (24). They were then dichotomized into 2 groups: those meeting recommended weekly amounts of physical activity and those not meeting recommended amounts of physical activity.

### Neighborhood characteristics

After defining a neighborhood as "the area within one-half mile or a 10-minute walk from your house," the 2004 Texas BRFSS asked respondents 6 state-added questions to evaluate their neighborhood built environment. Respondents rated the people in their neighborhood using 4 response options ranging from "very physically active" to "not at all physically active." They rated the neighborhood as a place to walk using 4 options ranging from "very pleasant" to "not at all pleasant." Respondents described the neighborhood street lighting by using 5 response options ranging from "very good" to "very poor" and the neighborhood safety by using 4 options ranging from "extremely safe" to "not at all safe." Respondents then indicated whether most people in the neighborhood can be trusted (yes/no) and whether the neighborhood has sidewalks (yes/no).

### Community facilities

The 2004 Texas BRFSS defined the community as a 5- to 10-mile drive from the respondent's house. Interviewers then asked respondents 5 questions to determine whether they used the following community recreational facilities for physical activity: private or membership-only facilities; walking trails, parks, playgrounds, and sports fields; shopping malls; public recreation facilities; and schools open for public recreation. For each, participants responded yes, no, or "My community does not have these facilities" (27).

### Analysis

We used descriptive statistics for the sample as a whole and by disability status. Given the sample size, distribution, and variance, we used logistic regression to determine the effect of the 6 predictors of neighborhood characteristics and 5 predictors of community facilities on the 2 dependent variables of LTPA and physical activity levels. We stratified predictors by disability status and analyzed them for LTPA and physical activity independently, controlling for race/ethnicity, age, education, and income, factors that are significant correlates of physical activity (28). Statistical differences were considered significant at α = .05. To conduct the analysis, we used SPSS version 17 (SPSS, Chicago, Illinois).

## Results

For the total study sample who met the inclusion criteria (N = 4,947), the mean age of participants was 42 years; 62% were female, 91% had graduated from high school, and 44% reported an annual income between $25,000 and $75,000 (Table 1). The total sample was racially and ethnically diverse; 58% were white and 30% were Hispanic.

Seventeen percent of respondents reported disabilities (n = 849). Of these, 60% reported LTPA outside of work (compared with 78% of those without disabilities) and 43% met recommended levels of physical activity (compared with 54% of respondents without disabilities). In general, people with disabilities perceived their neighborhoods less favorably and reported less use of community facilities for physical activity. To all questions regarding community facilities, people with disabilities more frequently reported that such facilities did not exist in their community (Table 1).

Both people with and without disabilities who reported using private recreation facilities (odds ratios [OR], 2.04 and 3.07, respectively) and trails, parks, playgrounds, or sports fields (OR, 2.12 and 2.36, respectively) were significantly more likely to report LTPA than those who reported no such use (Table 2). People with disabilities who described the street lighting for walking at night in their neighborhood as "very good" or "good" were less likely to participate in LTPA than those who described street lighting as "very poor" (OR, 0.34 and 0.50, respectively). However, the perceptions of people without disabilities about the condition of nighttime street lights proved unrelated to their LTPA. In contrast, the LTPA of people without disabilities was strongly associated with perceptions of neighborhood safety when participants classified their neighborhood as "extremely safe" (OR, 2.82), "quite safe" (OR, 3.05), or "slightly safe" (OR, 1.70) compared with "not at all safe" (Table 2).

For people with disabilities, the reported presence of neighborhood sidewalks, when compared with reported absence of sidewalks, was associated with meeting recommended PA levels (OR, 1.59) (Table 3). People with disabilities who reported their neighborhoods were "not very pleasant" places to walk (OR, 4.04) were more likely to meet physical activity recommendations than were people with disabilities responding that their neighborhoods were "not at all pleasant" places to walk. Furthermore, people with disabilities who reported their neighborhoods were "slightly safe" from crime, compared with "not at all safe," were less likely to meet physical activity recommendations (OR, 0.36). For people without disabilities, the use of private recreation facilities (OR, 1.47) and trails, parks, playgrounds, or sports fields (OR, 1.20) for physical activity was significantly related to meeting recommended levels of physical activity.

## Discussion

The use of walking trails, parks, playgrounds, or sports fields was significantly associated with the LTPA of people with disabilities. This study supports previous studies reporting that access to trails, parks, and playgrounds is associated with LTPA (2,3). Furthermore, a much greater proportion of people with disabilities reported that their community does not have these facilities or that they do not use them than did people without disabilities. This is probably the result of the facilities being inaccessible to people with disabilities, resulting in an unmet demand for access to trails, parks, playgrounds, and sports fields.

Among people with disabilities who reported LTPA, street lighting for walking at night was one of the neighborhood characteristics significantly related to reported LTPA, although the data indicate a negative association. No association was found between nighttime lighting and meeting recommended levels of physical activity for this demographic group. Furthermore, because the question about adequate street lighting specifically mentions walking, the results may have been different if other physical activities were examined. Although this finding is subject to confounding factors such as the definition of LTPA (eg, access to a car, daytime versus nighttime workout preferences, sidewalk conditions), it indicates that lighting is an important component of accessibility. People with disabilities using lighted areas at night are not using them as venues for physical activity but rather as nighttime accessible pathways.

The presence of neighborhood sidewalks is associated with meeting recommended levels of physical activity for people with disabilities. This finding coincides with the other issues already discussed related to accessibility. The presence and connectivity of sidewalks within a neighborhood not only provide venues for exercise but may also be the primary means of access to other facilities conducive to physical activity (17). Although parks, boulevards, boardwalks, and fitness facilities are still accessible to people without disabilities when sidewalks are lacking or poorly connected, people with disabilities are disproportionably affected (13,17).

LTPA was strongly associated with perception of neighborhood safety for people without disabilities, but people with disabilities did not show the same association. Yet, people with disabilities were more likely than people without disabilities to indicate that their neighborhood was unsafe. Chronic exposure to inaccessible and unsafe neighborhood environments may have conditioned people with disabilities to be less influenced by such conditions.

People with disabilities were less likely to participate in LTPA and meet recommendations for physical activity than people without disabilities (Table 1). These findings are consistent with those of other studies reporting inactivity prevalence among populations with disabilities (8), but actual LTPA and physical activity levels may differ from those reported here because of confounding factors such as self-reported activity, inaccuracies in memory recall, and the use of physical activity questions that may not adequately measure this demographic group (2,8). People with disabilities also were less likely to use any of the reported community facilities for physical activity except shopping malls, which are typically adequately accessible environments. In all instances, people with disabilities were more likely to report that the analyzed facilities did not exist in their community. This finding may indicate that people with disabilities are living in more dilapidated areas but more likely signifies a lack of accessibility and thus a functional unavailability of facilities (4).

As acknowledged by the Centers for Disease Control and Prevention, the use of BRFSS data in this circumstance creates some limitations. All information used in this study was gathered from surveys with a response rate below 50%. Further, the subjectivity of terms such as "pleasantness" and "safe" is vulnerable to respondent interpretation. However, the survey questions were designed to assess respondents' perceptions of their neighborhood. Studies to examine the validity of these perceptions in relation to actual environmental characteristics could further enhance the findings of this study. Likewise, findings might have differed greatly, as suggested elsewhere (2,8) if different questions were used that more adequately represented people with physical disabilities and more specifically analyzed their typical physical activities. For example, the Physical Activity and Disability Survey developed by Rimmer, Riley, and Rubin (30) allows people with disabilities more flexibility in describing their physical activity participation by acknowledging that commonly surveyed activities (eg, walking, bike riding, using private recreation facilities) might not accurately represent this population's physical activities. This study found significant differences between the LTPA and physical activity of people with and without disabilities. Results also indicate that the built environmental has similar effects on people with and without disabilities.

## Figures and Tables

**Table 1:** Participant Characteristics and Responses to Neighborhood and Physical Activity-Related Questions^a^ by Disability Status, Texas Behavioral Risk Factor Surveillance System, 2004

**Characteristic or Question/Answer**	Total Sample (N = 4,947)^b^	People With Disabilities (n = 849)^b^	People Without Disabilities (n = 4,098)^b^
**Mean age, y**	42	48	40
**Sex**			
Men	1,882 (38)	304 (36)	1,578 (39)
Women	3,065 (62)	545 (64)	2,520 (62)
**Education**			
Less than high school graduate	761 (15)	150 (18)	611 (15)
High school graduate or some college	2,554 (58)	484 (57)	2,070 (51)
College graduate	1,624 (33)	215 (25)	1,409 (34)
**Annual income, $**			
<25,000	1,481 (33)	339 (45)	1,142 (31)
25,000 to <75,000	1,971 (44)	312 (41)	1,659 (45)
≥75,000	1,000 (23)	111 (15)	889 (24)
**Race/ethnicity**			
White	2,836 (58)	560 (66)	2,276 (56)
Black	431 (9)	68 (8)	363 (9)
Hispanic	1,491 (30)	190 (23)	1,301 (32)
Other	164 (3)	27 (3)	137 (3)
**During the past month, other than your regular job, did you participate in any physical activities or exercise, such as running, calisthenics, golf, gardening, or walking for exercise?**			
Yes	3,711 (75)	515 (60)	3,196 (78)
No	1,233 (25)	333 (39)	900 (22)
**Met recommendations for moderate or vigorous physical activity.^c^**			
Yes	2,068 (52)	261 (43)	1,807 (54)
No	1,894 (48)	349 (57)	1,545 (46)
**In general, would you say that people in your neighborhood are . . .**			
Very physically active	501 (13)	87 (13)	414 (13)
Somewhat physically active	2,291 (58)	386 (56)	1,905 (58)
Not very physically active	869 (22)	150 (22)	719 (22)
Not at all physically active	314 (8)	65 (9)	249 (8)
**Overall, how would you rate your neighborhood as a place to walk?**			
Very pleasant	2,298 (54)	386 (51)	1,912 (55)
Somewhat pleasant	1,548 (36)	280 (37)	1,268 (36)
Not very pleasant	263 (6)	48 (6)	215 (6)
Not at all pleasant	157 (4)	39 (5)	112 (3)
**For walking at night, would you describe the lighting in your neighborhood as . . .**			
Very good	453 (11)	77 (10)	376 (11)
Good	1,124 (27)	181 (24)	943 (27)
Fair	1,164 (28)	194 (26)	970 (28)
Poor	816 (19)	152 (20)	664 (19)
Very poor	677 (16)	155 (20)	522 (15)
**How safe from crime do you consider your neighborhood to be? **			
Extremely safe	796 (19)	113 (13)	683 (20)
Quite safe	2,178 (51)	369 (49)	1,809 (52)
Slightly safe	994 (23)	196 (26)	798 (23)
Not at all safe	294 (7)	81 (11)	213 (6)
**Generally speaking, would you say most people in your neighborhood can be trusted?**			
Yes	3,400 (84)	572 (80)	2,828 (85)
No	644 (16)	143 (20)	501 (15)
**Does your neighborhood have sidewalks?**			
Yes	2,480 (58)	420 (55)	2,060 (58)
No	1,810 (42)	345 (42)	1,465 (42)
**Do you use any private or membership-only recreation facilities in your community for physical activity?**			
Yes	1,051 (25)	149 (18)	912 (26)
No	3,049 (71)	582 (77)	2,467 (70)
My community does not have these facilities	166 (4)	40 (5)	126 (4)
**Do you use walking trails, parks, playgrounds, or sports fields in your community for physical activity?**			
Yes	2,174 (51)	281 (37)	1,893 (54)
No	1,996 (47)	447 (58)	1,549 (44)
My community does not have these facilities	116 (2)	37 (5)	79 (2)

Abbreviation: CI, confidence interval.

a Participant information from adults aged 18 to 64 (29).

b All values presented as no. (%) except age.

c "Vigorous activities cause large increases in breathing or heart rate while moderate activities cause small increases in breathing or heart rate" (30).

**Table 2:** Likelihood of Adults Participating in Leisure-Time Physical Activity^a^^,^^b^ by Disability Status, Texas Behavioral Risk Factor Surveillance System, 2004 (N = 4,947)

Question/Answer	People With Disabilities (n = 849)		People Without Disabilities (n = 4,098)	
	Odds Ratio^c^ (95% CI)	*P* Value	Odds Ratio^c^ (95% CI)	*P* Value
**In general, would you say that people in your neighborhood are . . .**						
Very physically active	1.90 (0.86-4.21)	.11	0.96 (0.63-1.47)	.89
Somewhat physically active	2.26 (1.15-4.41)	.02	1.54 (1.09-2.18)	.02
Not very physically active	1.82 (0.89-3.74)	.10	1.28 (0.89-1.85)	.18
Not at all physically active	1 [Reference]		1 [Reference]	
**Overall, how would you rate your neighborhood as a place to walk?**						
Very pleasant	2.15 (0.87-5.33)	.10	1.40 (0.88-2.39)	.22
Somewhat pleasant	2.09 (0.86-5.10)	.11	1.15 (0.68-1.93)	.61
Not very pleasant	2.34 (0.82-6.69)	.11	1.11 (0.61-2.00)	.74
Not at all pleasant	1 [Reference]		1 [Reference]	
**For walking at night, would you describe the street lighting in your neighborhood as . . .**						
Very good	0.34 (0.16-0.72)	.005	0.76 (0.50-1.16)	.20
Good	0.50 (0.27-0.92)	.03	0.73 (0.53-1.02)	.07
Fair	0.78 (0.43-1.37)	.38	0.76 (0.55-1.05)	.10
Poor	0.77 (0.43-1.36)	.36	0.92 (0.66-1.28)	.63
Very poor	1 [Reference]		1 [Reference]	
**How safe from crime do you consider your neighborhood to be?**						
Extremely safe	1.82 (0.79-4.17)	.16	2.82 (1.78-4.48)	<.001
Quite safe	1.34 (0.67-2.71)	.41	3.05 (2.02-4.60)	<.001
Slightly safe	1.20 (0.61-2.34)	.61	1.70 (1.15-2.52)	.008
Not at all safe	1 [Reference]		1 [Reference]	
**Generally speaking, would you say most people in your neighborhood can be trusted?**						
Yes	1.05 (0.64-1.73)	.85	0.93 (0.70-1.23)	.60
No	1 [Reference]		1 [Reference]	
**Does your neighborhood have sidewalks?**						
Yes	1.12 (0.76-1.66)	.56	0.90 (0.73-1.10)	.31
No	1 [Reference]		1 [Reference]	
**Do you use any private or membership-only recreation facilities in your community for physical activity?**						
Yes	2.04 (1.23-3.40)	.006	3.07 (2.31-4.09)	<.001
My community does not have these facilities	0.53 (0.18-1.56)	.25	1.13 (0.63-2.02)	.68
No	1 [Reference]		1 [Reference]	
**Do you use walking trails, parks, playgrounds, or sports fields in your community for physical activity?**						
Yes	2.12 (1.41-3.18)	<.001	2.36 (1.91-2.92)	<.001
My community does not have these facilities	0.64 (0.18-2.31)	.50	0.92 (0.43-1.93)	.82
No	1 [Reference]		1 [Reference]	
**Do you use shopping malls in your community for physical activity and/or walking programs?**						
Yes	1.14 (0.72-1.82)	.58	0.74 (0.57-0.97)	.03
My community does not have these facilities	1.38 (0.55-3.51)	.49	0.87 (0.48-1.55)	.63
No	1 [Reference]		1 [Reference]	
**Do you use public recreation centers in your community for physical activity?**						
Yes	1.04 (0.63-1.73)	.88	1.15 (0.89-1.50)	.28
My community does not have these facilities	2.27 (0.57-9.03)	.25	1.10 (0.48-2.53)	.82
No	1 [Reference]		1 [Reference]	
**Do you use schools that are open in your community for public recreation activities?**						
Yes	1.01 (0.60-1.71)	.96	1.19 (0.93-1.52)	.17
My community does not have these facilities	1.76 (0.55-5.68)	.35	0.75 (0.33-1.70)	.49
No	1 [Reference]		1 [Reference]	

Abbreviation: CI, confidence interval.

a Participant information from adults aged 18 to 64 years (29).

b Participation in leisure-time physical activity determined by a yes response to the following question: "During the past month, other than your regular job, did you participate in any physical activities or exercise such as running, calisthenics, golf, gardening, or walking for exercise?" (29).

c Odds ratios adjusted for age, education, income, and race/ethnicity.

**Table 3:** Likelihood of Adults Meeting Recommended Levels of Moderate or Vigorous Physical Activity^a^^,^^b^ by Disability Status, Texas Behavioral Risk Factor Surveillance System, 2004 (N = 4,947)

Question/Answer	People With Disabilities (n = 849)		People Without Disabilities (n = 4,098)	
	Odds Ratio^c^ (95% CI)	*P* Value	Odds Ratio^c^ (95% CI)	*P* Value
**In general, would you say that people in your neighborhood are . . .**				
Very physically active	1.64 (0.61-4.40)	.32	1.38 (0.93-2.06)	.11
Somewhat physically active	1.50 (0.63-3.58)	.36	1.03 (0.74-1.44)	.86
Not very physically active	1.89 (0.75-4.78)	.18	1.01 (0.71-1.45)	.94
Not at all physically active	1 [Reference]		1 [Reference]	
**Overall, how would you rate your neighborhood as a place to walk?**				
Very pleasant	1.51 (0.49-4.64)	.47	1.60 (0.95-2.69)	.08
Somewhat pleasant	1.87 (0.61-5.69)	.27	1.36 (0.81-2.27)	.24
Not very pleasant	4.04 (1.03-15.84)	.045	1.20 (0.68-2.12)	.54
Not at all pleasant	1 [Reference]		1 [Reference]	
**For walking at night, would you describe the street lighting in your neighborhood as . . .**				
Very good	1.82 (0.76-4.20)	.16	0.92 (0.65-1.30)	.62
Good	0.97 (0.50-1.90)	.93	0.75 (0.57-0.99)	.04
Fair	0.86 (0.46-1.62)	.65	0.77 (0.59-1.01)	.06
Poor	0.72 (0.38-1.35)	.30	0.88 (0.67-1.17)	.37
Very poor	1 [Reference]		1 [Reference]	
**How safe from crime do you consider your neighborhood to be?**				
Extremely safe	0.59 (0.22-1.56)	.29	1.03 (0.65-1.64)	.90
Quite safe	0.54 (0.23-1.27)	.16	0.93 (0.60-1.44)	.74
Slightly safe	0.36 (0.16-0.84)	.02	1.04 (0.88-1.24)	.63
Not at all safe	1 [Reference]		1 [Reference]	
**Generally speaking, would you say most people in your neighborhood can be trusted?**				
Yes	1.40 (0.79-2.48)	.26	1.03 (0.79-1.34)	.86
No	1 [Reference]		1 [Reference]	
**Does your neighborhood have sidewalks?**				
Yes	1.59 (1.02-2.46)	.04	1.04 (0.88-1.24)	.63
No	1 [Reference]		1 [Reference]	
**Do you use any private or membership-only recreation facilities in your community for physical activity?**				
Yes	1.53 (0.94-2.50)	.09	1.47 (1.22-1.76)	<.001
My community does not have these facilities	1.40 (0.40-4.94)	.60	1.11 (0.66-1.86)	.70
No	1 [Reference]		1 [Reference]	
**Do you use walking trails, parks, playgrounds, or sports fields in your community for physical activity?**				
Yes	1.25 (0.82-1.92)	.30	1.20 (1.01-1.43)	.04
My community does not have these facilities	1.79 (0.41-7.72)	.44	1.71 (0.83-3.52)	.15
No	1 [Reference]		1 [Reference]	
**Do you use shopping malls in your community for physical activity and/or walking programs?**				
Yes	0.67 (0.41-1.16)	.17	0.77 (0.61-0.96)	.02
My community does not have these facilities	0.72 (0.25-2.10)	.55	0.82 (0.50-1.34)	.43
No	1 [Reference]		1 [Reference]	
**Do you use public recreation centers in your community for physical activity?**				
Yes	1.02 (0.61-1.72)	.93	1.07 (0.88-1.29)	.52
My community does not have these facilities	2.05 (0.45-9.30)	.35	0.88 (0.44-1.80)	.74
No	1 [Reference]		1 [Reference]	
**Do you use schools that are open in your community for public recreation activities?**				
Yes	1.61 (0.92-2.81)	.10	1.17 (0.97-1.41)	.11
My community does not have these facilities	0.49 (0.13-1.83)	.29	0.93 (0.45-1.94)	.85
No	1 [Reference]		1 [Reference]	

Abbreviation: CI, confidence interval.

a Participant data for adults aged 18 to 64 (29).

b Respondents were determined to meet recommended weekly levels of physical activity if they reported engaging in 20 minutes or more of vigorous activity on at least 3 days or 5 days with 30 minutes of moderate activity (24).

c Odds ratios adjusted for age, education, income, and race/ethnicity.
