# Erratum, Vol. 7: Issue 5

**Published:** 2010-12-15

In the article "Effects of Perceived Neighborhood Characteristics and Use of Community Facilities on Physical Activity of Adults With and Without Disabilities," reference 30 was cited as reference 31 in the text. The correction was made to our website on September 07, 2010, and appears online at http://www.cdc.gov/pcd/issues/2010/sep/09_0179.htm. We regret any confusion or inconvenience this error may have caused.

En el artículo "Efectos de la percepción de las características del vecindario y del uso de las instalaciones comunitarias en la realización de actividad física en adultos con o sin discapacidades", la referencia bibliográfica 30 fue citada en el texto como referencia bibliográfica 31. La corrección se hizo en nuestro sitio web el 7 de septiembre del 2010, y se puede encontrar en línea en http://www.cdc.gov/pcd/issues/2010/sep/09_0179_es.htm. Lamentamos cualquier inconveniente o confusión que este error haya podido causar.

